# Chemical Composition of Salmon Ovary Outer Membrane and Its Protein Increases Fecal Mucins Content in C57BL/6J and Type 2 Diabetic/Obese KK-*A^y^* Mice

**DOI:** 10.3390/foods2030415

**Published:** 2013-09-06

**Authors:** Hayato Maeda, Ryota Hosomi, Utako Chiba, Kenji Fukunaga

**Affiliations:** 1 Faculty of Agriculture and Life Science, Hirosaki University, Hirosaki, Aomori 036-8561, Japan; E-Mails: hayatosp@cc.hirosaki-u.ac.jp (H.M.); uta_f28@yahoo.co.jp (U.C.); 2 Division of Food Science and Nutrition, Tottori College, Kurayoshi, Tottori 682-8555, Japan; 3 Faculty of Chemistry, Materials and Bioengineering, Kansai University, Suita, Osaka 564-8680, Japan; E-Mail: fukunagk@kansai-u.ac.jp

**Keywords:** salmon ovary outer membrane, byproducts, collagen, mucins, resistant protein, fecal ammonia, protein digestibility

## Abstract

Salmon ovary outer membrane (SOM) is a byproduct of the salmon industry; however, the effective utilization of SOM for food materials and supplements is anticipated as the demand for fish and seafood increases worldwide. The purposes of the present study were to assess the chemical composition of SOM, the characteristics of SOM protein (SOMP), and its effects on serum and fecal biochemical parameters in mice. SOM contained high levels of crude protein (61.9 g/100 g) and crude lipid (18.9 g/100 g). The protein pattern of SOMP was different from those of fish muscle protein and roe; it was abundant in collagen, as calculated from the hydroxyproline content. In addition, SOMP exhibited lower protein digestibility during *in vitro* digestion analyses compared with casein. Male C57BL/6J and KK-*A^y^* mice were fed a casein-based semi-purified diet or a diet with replacement of part of the dietary protein (50%) by SOMP for four weeks. Mice fed the diet containing SOMP showed elevated fecal nitrogen and mucins contents and reduced levels of serum liver injury markers and fecal ammonia. These results show for the first time that chemical composition of SOM, and SOMP, contain a resistant protein fraction and a large amount of collagen. Therefore, SOM is a potential source of marine collagen and functional food material for promoting the health of the liver and colon.

## 1. Introduction

Recently, the production of salmon meat and processed food has increased both in Japan and worldwide [1]. Salted chum salmon (*Oncorhynchus keta*) roe product, named ikura, has been consumed as a traditional food in Japan. Salmon roe, from the interior of an ovary, contains large amounts of proteins and lipids, such as *n*-3 polyunsaturated fatty acids (PUFAs), including eicosapentaenoic acid (EPA) and docosahexaenoic acid (DHA). Previous researches demonstrated that salmon roe lipids prevented chronic liver disease [2], and phospholipids extracted from salmon roe reduced plasma triacylglycerol (TG) and cholesterol contents in rats [3]. In addition, a few studies have suggested that salmon roe protein can inhibit angiotensin-converting enzyme [4].

Ikura is removed from the ovary sack before it is salted; thus, the salmon ovary outer membrane (SOM), namely, the ovary sack, has been treated as a waste product. However, the issue of seafood byproduct disposal has become a concern as the production of fish and seafood has increased [5]. Therefore, the effective usage of salmon byproducts, such as bone, head, and ovary outer membrane, has been anticipated. Some salmon byproducts are utilized as protein sources for animal feed [6]. From an economic perspective, salmon industry byproducts should be used for food, supplements, and cosmetic products for humans rather than be used for the feed industry. However, no information is available concerning the chemical composition and health-promoting effects of SOM. SOM is removed from the ovary sack during ikura preparation in a food factory. In this process, SOM is produced as a waste product. However, SOM is disposed of a sanitary manner by hand, its conversion to a food material is easy compared with other waste products. Upon measuring the nutrient composition of SOM, we found that a large amount of protein is contained in it. Fish protein is a high quality source of human protein, and has been reported to reduce serum cholesterol levels [7] and suppress chemically induced colon cancer [8]. Collagen from marine sources has been paid as alternative collagen sources due to the occurrence of bovine spongiform encephalopathy, transmissible spongiform encephalopathy, and the foot-and-mouth disease crisis [9]. To clarify the possible usage of SOM as a potential source of collagen and health-promoting food material, we investigated the chemical composition of SOM and the profile of protein obtained from SOM.

Obesity and being overweight has been identified as a multiple risk factor for diabetes, cardiovascular disease, and some cancers [10]. This issue becomes important, not only medically, but also socioeconomically [11]. Therefore, we also demonstrated the effects of dietary SOM protein (SOMP) on the biochemical compositions of serum and feces in C57BL/6J and type 2 diabetic/obese KK-*A^y^* mice fed a high fat diet. KK-*A^y^* mouse, a genetic animal model of non-insulin-dependent diabetes mellitus, exhibits hyperglycemia, polyuria, and polydipsia due to overfeeding [12]. We believe that the usage of salmon byproducts is important for the conservation of aquatic species as the demand for fish and seafood has increased worldwide.

## 2. Experimental Section

### 2.1. Materials

Chum salmon (*O. keta*) ovary outer membrane was obtained in November, 2011, from Hachinohe Kanzume Co., Ltd. (Aomori, Japan). The ingredients of an animal diet were obtained from Oriental Yeast Co., Ltd. (Tokyo, Japan). All other chemicals were of reagent grade and were obtained from commercial sources.

### 2.2. Preparation of Salmon Ovary Outer Membrane Protein

SOM (moisture 87.7% w/w) was washed with cold distilled water and then freeze-dried. The dried material was washed with *n*-hexane to remove protein-associated lipids. The residual product was freeze-dried, ground using a mill (GM200; Retsch Co., Ltd., Dusseldorf, Germany) and then stored at −30 °C. The resulting product was called SOMP. The yield rate for SOMP was 9.4% (w/w) in terms of its preparation from SOM.

### 2.3. Chemical Analysis

The chemical composition of SOM was measured in accordance with the AOAC methods [13]. The protein contents were estimated to use a nitrogen-to-protein conversion factor of 5.55 [14].

Lipid was extracted in accordance with the method of Bligh and Dyer [15]. Phospholipid (PL) content was measured by phosphorus analysis [16]. Cholesterol content was analyzed by gas-liquid chromatography (GC) (Shimadzu Co., Kyoto, Japan) using a packed-column with silicone SE-30 (Shinwa Chemical Industries Ltd., Kyoto, Japan), with an internal standard. The fatty acid composition was determined by analysis using an Omegawax 250 column (Sigma-Aldrich Japan Co. Ltd., Tokyo, Japan) in the GC system (Shimadzu Co.) after methylation with sodium methoxide [17]. The initial oven temperature of 120 °C was increased at 2 °C/min to 240 °C. The temperatures at injection and detection were 250 °C and 260 °C, respectively. The identification of each fatty acid species was carried out using standard mixture of key fatty acid methyl ester (Supelco^®^ 37 Component FAME Mix, Sigma-Aldrich Japan Co. Ltd.).

For amino acid analysis, the SOMP and casein were hydrolyzed by 6 M HCl at 110 °C for 24 h. The amino acid compositions of SOMP and casein were measured using high-performance liquid chromatography with UV detection derived by phenylisothiocyanate [18]. Each amino acid was identified using the retention time of commercially available authentic standard mixtures.

### 2.4. Sodium Dodecyl Sulfate Polyacrylamide Gel Electrophoresis (SDS-PAGE) Analysis

The molecular weight distribution was analyzed by sodium dodecyl sulfate polyacrylamide gel electrophoresis (SDS-PAGE), which was carried out in accordance with the methods of Scaggerr and von Jagow [19], with slight modifications. The protein solution was mixed with Laemmli sample buffer and then heated 95 °C for 5 min [20]. Aliquots (5 µg) of the samples were loaded on a 4% polyacrylamide stacking gel, and then separated in a 12.5% polyacrylamide separation gel, together with a protein molecular weight marker (SDS-PAGE Molecular Weight Standard, Broad Range; Bio-Rad Laboratories, Inc., CA, USA). The resulting protein bands were stained with 0.25% (w/v) coomassie brilliant blue G in 45% methanol and 10% acetic acid for 1 h. Then, staining gel was destained using 25% methanol and 10% acetic acid.

### 2.5. *In Vitro* Digestion

SOMP and casein were digested in accordance with the gastrointestinal digestion method of Hosomi *et al*. [7] with slight modifications. Hydrolysis parameters of pepsin were as follows: temperature, 37 °C; pH, 2.0; enzyme/substrate ratio, 1:100 (w/w); protein isolate concentration, 10% (w/v); pH was adjusted with HCl. After incubation for 120 min under 100 rpm agitation, protein hydrolysis solution was neutralized with NaOH for inactivation of pepsin. Porcine pancreatin was added; pancreatin hydrolysis parameters were as follows: temperature, 37 °C; pH, 7.4; enzyme/substrate ratio, 1:30 (w/w); pH was adjusted with NaOH. After incubation for 120 min under 100 rpm agitation, digestion was inactivated by boiling for 15 min. The residual product was washed with cold distilled water three times and centrifuged at 4500× *g* at 4 °C for 15 min, freeze-dried, and then weighed.

### 2.6. Protein Digestibility

For the monitoring of protein digestibility at intervals during digestion, aliquots of the digestion reaction were removed at 0, 2, 5, 10, 15, 20, 30, 60, and 120 min during the pepsin and pancreatin *in vitro* digestion. The aliquots were mixed with an equal volume of 24% trichloroacetic acid for inactivating the pepsin and pancreatin, and then centrifuged at 7500× *g* at 4 °C for 10 min. Protein digestibility was calculated using the measurement of free amino groups, due to the reaction with 2,4,6-trinitrobenzene sulfonic acid [21]. L-Leucine was used as a standard in the protein digestibility assays.

### 2.7. Animal Diet and Care

The animal experimental protocol was approved and reviewed by the committee of Hirosaki University (Aomori, Japan). C57BL/6J (male, 3 weeks old) and KK-*A^y^* mice (male, 3 weeks old) were purchased from Charles River Laboratories Japan, Inc. (Ibaraki, Japan) and CLEA Japan, Inc. (Tokyo, Japan), respectively. The animals were kept in an air-controlled room (temperature, 20–21 °C; humidity, 60%–65%; lights on, 07:00–19:00). C57BL/6J and KK-*A^y^* mice were acclimatized for one week with *ad libitum* access to tap water and control diets that were produced according to the AIN93G formula [22]. The compositions of experimental diets are presented in Table 1. After acclimation, C57BL/6J and KK-*A^y^* mice were divided into two groups of seven mice, each with similar mean body weights.

Daily the food and water consumption, and body weight was recorded. For one week before sacrifice, feces were collected from each group, every 24 h. After the mice were fed diets for 4 weeks, the mice were weighed and sacrificed while being anesthetized with diethyl ether. The mice were not deprived of food prior to being sacrificed because genes related to lipid metabolism were significantly down-regulated by fasting [23]. Blood was drawn from the descending part of abdominal aorta without anti-coagulants, and blood was centrifuged at 2000× *g* for 15 min for separation of serum. Liver, kidney, spleen, brown adipose tissue (BAT), and epididymal, perirenal, and retroperitoneal white adipose tissue (WAT), were removed rapidly, weighed, washed with cold saline, and then frozen using liquid nitrogen, followed by storage at −70 °C until analysis.

**Table 1:** Composition of the experimental diets.

Composition	C57BL/6J		KK-*A^y^*	
	Control	SOMP	Control	SOMP
	g/kg			
Casein ^†^	258	129	230	115
Salmon ovary outer membrane protein ^‡^	-	129	-	115
Dextrinized corn starch	60.2	60.2	92.1	92.1
Corn starch	181.286	181.286	277.386	277.386
Sucrose	100	100	100	100
Cellulose	50	50	50	50
AIN93G mineral mixture	35	35	35	35
AIN93 vitamin mixture	10	10	10	10
l-Cystine	3	3	3	3
Choline bitartrate	2.5	2.5	2.5	2.5
Lard	230	230	130	130
Soybean oil	70	70	70	70
*tert*-Butylhydroquinone	0.014	0.014	0.014	0.014

^†^ The composition of casein was as follows (% w/w): protein, 87.5; fat, 1.5; moisture, 7.2; and ash, 1.7; ^‡^ The composition of salmon ovary outer membrane protein was as follows (% w/w): protein, 86.9; fat, 1.2; moisture, 8.0; and ash, 1.6.

### 2.8. Analysis of Serum and Fecal Biochemical Compositions

Serum TG, non-esterified fatty acid (NEFA), PL, cholesterol, aspartate aminotransferase (AST), and alanine aminotransferase (ALT) were measured by automatic analyzer (AU5431, Olympus Co., Tokyo, Japan).

Collected feces were freeze-dried and then ground to a fine powder. Fecal fatty acid content was measured in accordance with the method of van de Kamer *et al*. [24]. Nitrogen content was estimated according to the Kjeldahl method. Ammonia was assayed in fecal suspension, which was centrifuged at 1500× *g* at 4 °C for 15 min, using a colorimetric kit (Ammonia-Test-Wako kit; Wako Pure Chemical Industries Ltd., Osaka, Japan) after deactivation of endogenous urease activity at 80 °C for 15 min. Mucins were determined by a specific fluorimetric assay, and *N*-acetylgalactosamine was used as standard [25].

### 2.9. Statistical Analysis

Data represent means and standard deviations (SD). The statistical significant differences were estimated by Student’s *t*-test. Significant difference was considered at *p* value of <0.05. Statistical analysis was carried out by StatView-J software, version 5.0 (Abacus Concepts, CA, USA).

## 3. Results and Discussion

### 3.1. Chemical Composition of SOM and SOMP

The composition of SOM is presented in Table 2. SOM was found to contain high levels of crude proteins (61.9 g/100 g) and crude lipids (18.9 g/100 g). The lipid of SOM mainly consisted of TG (40.1% of total lipids), PL (52% of total lipids), and total cholesterol level in the SOM lipids was as high (5.8% of total lipids) as that of salmon roe. The main fatty acids of the SOM lipids were as follows (% w/w): DHA, 18.8%; EPA, 11.1%; palmitic acid, 14.7%; and oleic acid, 16.8%. The beneficial health-promoting effects of the *n*-3 PUFAs involving EPA and DHA have been demonstrated extensively [26]. In addition, protein extracted from various fish species and parts also has many health-promoting effects, such as improved blood pressure [27], stimulated fibrinolytic activity [28], and hypocholesterolemia [7]. There is a potential that the protein of SOM has health-promoting and -maintaining effects, thus, in this study, SOMP was prepared from SOM. The composition of casein, which is the protein source of the basal diet (AIN93G) in the animal experiment, was as follows (% w/w): crude protein, 87.5; crude lipid, 1.5; moisture, 7.2; and ash, 1.7.

**Table 2:** Chemical composition of salmon ovary outer membrane (SOM) and salmon ovary outer membrane protein (SOMP).

Composition			SOM ^†^	SOMP
Crude protein (g/100 g)			61.9	86.9
Crude lipid (g/100 g)			18.9	1.2
	Triacylglycerol (g/100 g lipid)		40.1	-
	Phospholipids (g/100 g lipid)		52.0	-
	Total cholesterol (g/100 g lipid) ^‡^		5.8	-
	Fatty acid composition (%)			
		C16:0	14.7	-
		C16:1 *n*-9	5.1	-
		C18:0	3.4	-
		C18:1 *n*-9	16.8	-
		C20:4 *n*-6	1.2	-
		C20:5 *n*-3 (EPA)	11.1	-
		C22:6 *n*-3 (DHA)	18.8	-
Moisture (g/100 g)			7.1	8.0
Ash (g/100 g)			5.9	1.6

^†^ SOM was freeze-dried and then ground to a fine powder; ^‡^ Total cholesterol was sum of free cholesterol and cholesterol ester; EPA, eicosapentaenoic acid; DHA, docosahexaenoic acid; SOM, salmon ovary outer membrane; SOMP, salmon ovary outer membrane protein.

### 3.2. Amino Acid Composition and Molecular Weight of SOMP

The amino acid compositions of SOMP and casein are presented in Table 3. SOMP, compared with casein, was rich in arginine, aspartic acid, glycine, and threonine, whereas the levels of glutamic acid and proline were low. The major characteristics of the amino acid composition of SOMP were high levels of hydroxyproline and glycine. Hydroxyproline and glycine are major components of collagen, and the collagen content was calculated by multiplying the hydroxyproline content by 11.42 [29]. SOMP was shown to contain a large amount of collagen (708 g/kg protein) as calculated by the hydroxyproline content. Collagen has a wide range of applications in food industries, cosmetic, pharmaceutical, biomedical, and tissue engineering due to its great biocompatibility and biodegradability [30]. Recently, collagen from marine sources has been receiving increasing attention as alternative sources for the replacement of mammalian collagen. Therefore, SOM can be used as a potential source for marine collagen extraction.

**Table 3:** Amino acid composition of SOMP.

Amino acid	SOMP	Casein
	g/kg protein	
Alanine	60	44
Arginine	72	28
Aspartic acid ^†^	118	68
Glutamic acid ^‡^	114	184
Glycine	104	31
Histidine	23	26
Hydroxyproline	62	ND
Isoleucine	32	54
Leucine	46	92
Lysine	59	69
Methionine	21	25
Phenylalanine	39	39
Proline	50	123
Serine	52	62
Threonine	79	44
Tyrosine	33	39
Valine	35	72

^†^ Aspartic acid: aspartic acid + asparagine; ^‡^ Glutamic acid: glutamic acid + glutamine; ND, not detected; SOMP, salmon ovary outer membrane protein.

**Figure 1:**
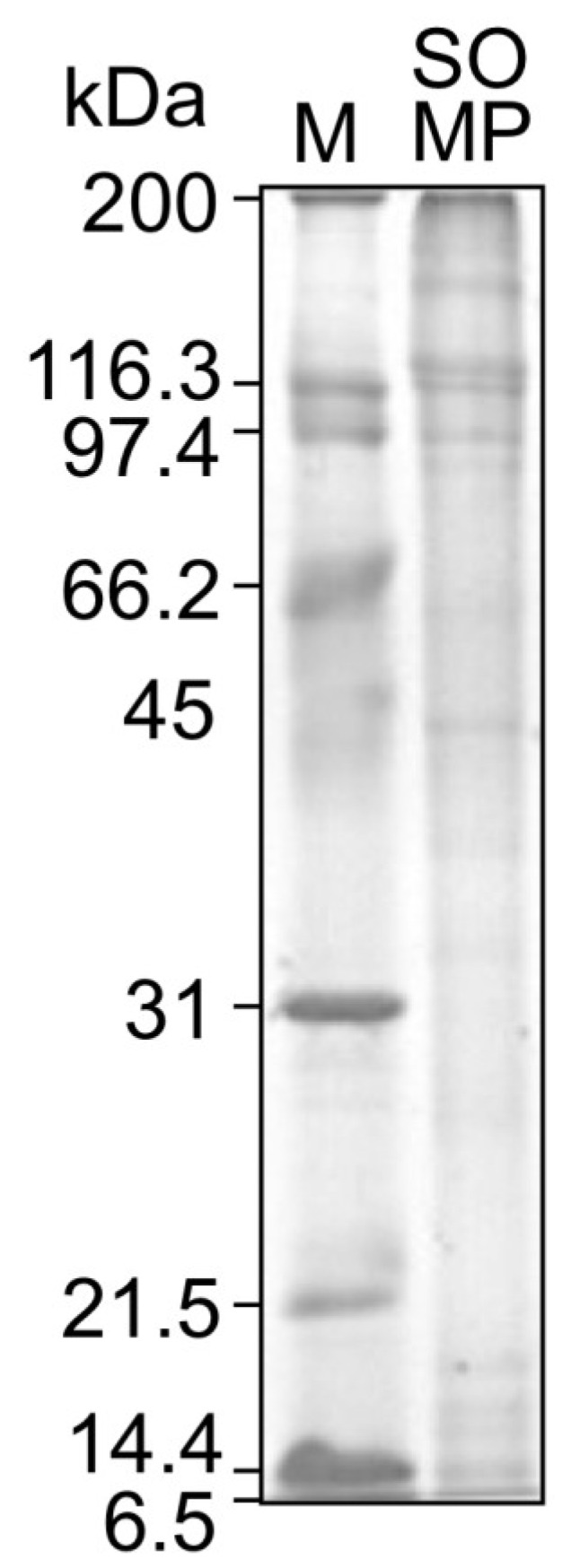
SDS-PAGE patterns of SOMP.

The molecular weight of SOMP is presented in Figure 1. The SOMP included a number of bands ranging from 200 to 97.4 kDa. Two prominent bands of around 116.3 kDa could be α-1 and α-2 collagen chains, as described in previous research [31]. There are, at least, over 28 types of collagen from various animal tissues and each type has a molecular structure and distinctive amino acid sequence [31]. Further studies are required to clarify which type of collagen SOM. The results demonstrated that the molecular weight profile of SOMP was largely different from that of salmon roe protein and popular fish muscle proteins, such as Alaska pollock, which contains large amounts of myosin (light chain, 21 kDa) and actin (approximately 45 kDa) [32,33].

SDS-PAGE was performed using 12.5% polyacrylamide separation gel; Lane (M), molecular weight marker; Lane (SOMP), salmon ovary outer membrane protein.

### 3.3. *In Vitro* Digestion of SOMP

**Figure 2:**
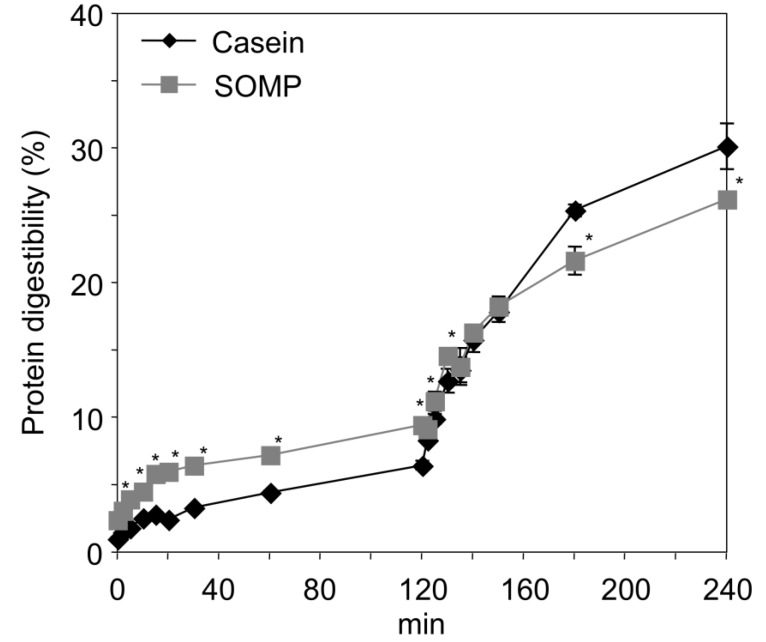
Course of protein digestibility of casein and SOMP using pepsin (added at time 0 min) and pancreatin (added at time 120 min).

The time courses of protein digestibility of SOMP and casein are presented in Figure 2. The levels of protein digestibility of casein and SOMP, by pepsin, were high in the first 20 min, after which digestion proceeded slowly, and the protein digestibility of SOMP was significantly higher than that of casein from 5 min to 120 min. During pancreatin digestion, the levels of protein digestibility of casein and SOMP were dramatically increased, and the protein digestibility of SOMP was significantly lower than that of casein from 180 min to 240 min. SOMP had a significantly higher insoluble sediment production rate, after the *in vitro* digestion, compared with casein (casein and SOMP: 1.2% ± 0.1% w/w and 5.8% ± 0.4% w/w, respectively). A previous *in vitro* study showed that collagen was adequately digested by pepsin and pancreatic enzymes [34]. From these results, SOMP could contain resistant proteins that are undigested remnants of dietary proteins, and these resistant proteins might be another protein fraction besides collagen in SOMP. Resistant proteins, having the same function as fiber in the intestine [35], have been shown to have beneficial effects via the enhancement of fecal bile acids and nitrogen excretion [36], and the suppression of colon carcinogenesis [37]. Therefore, in the next experiment, C57BL/6J and KK-*A^y^* mice were fed a diet containing SOMP for 4 weeks, after which the effects of this diet were examined.

Each point represents means ± SD (*n* = 3); Values are significantly different compared with the casein at * *p* < 0.05 using Student’s *t*-test; SOMP, salmon ovary outer membrane protein.

### 3.4. Effects of SOMP on Growth Parameters and Organ Weights

The growth parameters and relative organ weights are presented in Table 4. The food intake of KK-*A^y^* mice is higher than that of C57BL/6J mice, which is concomitant with increases of final body weight and body weight gain. However, no influence of SOMP administration on the growth parameters was observed.

There were no significant differences in relative weights of liver, kidney, spleen, epididymal WAT, and perirenal and retroperitoneal WAT, between the control and SOMP groups in C57BL/6J mice. Heart, kidney, jejunum, liver, and spleen weights gained in KK-*A^y^* mice due to the accumulation of fat [38]. However, no influence of SOMP administration on relative organ weights in KK-*A^y^* mice was observed.

**Table 4:** Effect of dietary SOMP on growth parameters and relative organ weights in C57BL/6J and KK-*A^y^* mice.

	C57BL/6J		KK-*A^y^*	
	Control	SOMP	Control	SOMP
*Growth parameters*				
Initial BW (g)	18.0 ± 0.7	18.2 ± 0.5	18.1 ± 1.9	18.4 ± 2.1
Final BW (g)	28.2 ± 1.9	26.9 ± 1.4	43.5 ± 2.0	42.2 ± 5.4
BW gain (g/day)	0.28 ± 0.07	0.25 ± 0.05	0.91 ± 0.33	0.85 ± 0.17
Food intake (g/day)	3.41 ± 0.65	3.15 ± 0.68	5.70 ± 0.35	5.39 ± 0.32
Food efficiency (g/g) ^†^	0.082 ± 0.018	0.080 ± 0.016	0.107 ± 0.010	0.114 ± 0.030
Water intake (mL/day)	2.94 ± 1.14	3.31 ± 1.28	35.0 ± 11.8	35.0 ± 11.8
*Relative organ weight (g/100 g BW)*				
Liver weight	4.01 ± 0.30	3.94 ± 0.27	5.66 ± 0.45	5.85 ± 0.39
Kidney	1.19 ± 0.09	1.27 ± 0.06	1.53 ± 0.11	1.54 ± 0.13
Spleen	0.24 ± 0.03	0.24 ± 0.02	0.29 ± 0.06	0.26 ± 0.03
Epididymal WAT	3.99 ± 0.76	3.87 ± 0.42	4.88 ± 0.42	4.66 ± 0.40
Perirenal and retroperitoneal WAT	1.19 ± 0.17	1.33 ± 0.20	2.22 ± 0.38	2.20 ± 0.27
BAT	0.48 ± 0.04	0.45 ± 0.04	0.74 ± 0.11	0.67 ± 0.10

Data represent means ± SD (*n* = 7); Values are significantly different compared with the control group of each mouse at ^*^p < 0.05 using Student’s *t*-test; ^†^ Food efficiency (g/g) = BW gain (g/day)/food intake (g/day); BAT, brown adipose tissue; BW, body weight; WAT, white adipose tissue.

### 3.5. Effects of SOMP on Serum and Fecal Biochemical Parameters

The serum and fecal biochemical parameters are presented in Table 5. In C57BL/6J and KK-*A^y^* mice, there were no significant differences in serum TG, NEFA, PL, and cholesterol contents between the control and SOMP groups. The serum ALT level was significantly lower in the SOMP group than in the control group, and the serum AST level in the SOMP group tended to be lower than in the control group (*p* = 0.09) in KK-*A^y^* mice. Serum AST and ALT levels are liver injury markers. KK-*A^y^* mice develop steatohepatitis spontaneously due to insulin resistance, which resembles some pathological features of human non-alcoholic steatohepatitis [39]; thus, the serum AST and ALT levels were increased compared with those in C57BL/6J mice [40]. Dietary SOMP may have a protective effect against the liver injury associated with metabolic syndrome in KK-*A^y^* mice. However, SOMP diet did not influence the diabetic symptoms such as hypertriglyceridemia in KK-*A^y^* mice.

Fecal dry weights in C57BL/6J and KK-*A^y^* mice were not affected by SOMP administration. In C57BL/6J mice, the SOMP diet decreased fatty acid excretion in feces, while this was not altered in KK-*A^y^* mice. The SOMP diet was also associated with significantly higher fecal nitrogen excretion than the control diet in C57BL/6J and KK-*A^y^* mice. As for the results of *in vitro* digestion and animal experiments, SOMP includes resistant proteins. Resistant proteins are indigestible remnants of dietary protein, and thus are associated with the problem of increased protein fermentation products, including ammonia and polyamines. A previous study showed that high ammonia content in feces was cytotoxic and may promote carcinogenesis in the colon [41]. In this study, the SOMP group of KK-*A^y^* mice exhibited lower fecal ammonia content than the control group. In addition, fecal mucins excretion was significantly enhanced by dietary SOMP in both C57BL/6J and KK-*A^y^* mice. Previous studies suggested that the consumption of sericin and burdock, containing resistant proteins, elevated fecal mucins levels [42,43]. Hence, the dietary SOMP effectively promoted the secretion of mucins in the small intestine due to it containing resistant proteins. Mucins are main components of the initial barrier of host defense against pathogens in intestines [44]. High intestinal mucins production has been associated with colon cancer risk reduction [45]. Therefore, there is a possibility that dietary SOMP can protect against colon carcinogenesis through the suppression of ammonia fermentation and the enhancement of mucins secretion. However, further research is necessary to prove this hypothesis. The results obtained in this study suggest that the consumption of SOMP has beneficial effects on the intestinal environment and barrier function in C57BL/6J and KK-*A^y^* mice fed a high-fat diet.

**Table 5:** Effect of dietary SOMP on serum and fecal biochemical parameters in C57BL/6J and KK-*A^y^* mice.

	C57BL/6J						KK- *A^y^*					
	Control			SOMP			Control			SOMP		
*Serum*												
TG (mg/dL)	51.3 ± 29.5			44.6 ± 9.4			649 ± 320			457 ± 158		
NEFA (μEq/L)	1866 ± 520			1881 ± 353			4034 ± 1052			3691 ± 1064		
PL (mg/dL)	345 ± 27			318 ± 22			371 ± 34			384 ± 50		
Cholesterol (mg/dL)	177 ± 13			168 ± 13			182 ± 18			196 ± 19		
AST (IU/L)	-						121.1 ± 48.4			87.6 ± 13.7		
ALT (IU/L)	-						42.5 ± 4.5			34.0 ± 2.9 *		
*Feces*												
Dry weight (g/day)	1.75 ± 0.16			1.63 ± 0.18			2.67 ± 0.77			2.84 ± 0.78		
Fatty acids (mg/day)	0.81 ± 0.06			0.50 ± 0.06 *			0.16 ± 0.04			0.23 ± 0.08		
Nitrogen (mg/day)	35.1 ± 4.7			43.0 ± 7.0 *			47.2 ± 7.0			72.3 ± 17.1 *		
NH_3_ (mg/day)	2.39 ± 0.68			2.58 ± 0.33			3.48 ± 0.70			2.40 ± 0.71 *		
Mucins (μg/day)	119 ± 30			174 ± 54 *			186 ± 42			269 ± 75 *		

Data represent means ± SD (*n* = 7); Values are significantly different compared with the control group of each mouse at * *p* < 0.05 using Student’s *t*-test; ALT, alanine aminotransferase; AST, aspartate aminotransferase; NEFA, non-esterified fatty acid; PL, phospholipids; TG, triacylglycerol.

## 4. Conclusions

This study, for the first time, showed that SOM contains a high level of protein (61.9% w/w), and SOMP has abundant collagen, at about 708 g/kg protein, as calculated from the hydroxyproline content. According to *in vitro* digestion and animal experiments, SOMP shows low digestibility due to mammalian digestive enzymes, and thus contains resistant proteins. The consumption of SOMP can decrease serum liver injury markers and fecal ammonia content, and increase fecal mucins content; thus, it might have health-promoting effects on liver and colon. From this study, it can thus be concluded that SOM, which is a salmon byproduct, can be used for potential source of marine collagen and functional food materials.
